# Interaction between ω-6 fatty acids intake and blood cadmium on the risk of low cognitive performance in older adults from National Health and Nutrition Examination Survey (NHANES) 2011–2014

**DOI:** 10.1186/s12877-022-02988-7

**Published:** 2022-04-07

**Authors:** Guangxiang Huang, Gang Ren

**Affiliations:** 1 Department of Neurology, The First People’s Hospital of Xiaoshan District, Xiaoshan First Affiliated Hospital of Wenzhou Medical University, 311200 Hangzhou, Zhejiang P. R. China; 2 Department of Neurology, Shanxi Bethune Hospital, Shanxi Academy of Medical Sciences, Tongji Shanxi Hospital, Third Hospital of Shanxi Medical University, No.99 Longcheng Street, Shanxi 030032 Taiyuan, P. R. China

**Keywords:** ω-6 fatty acids, Blood cadmium, Low cognitive performance, Interaction

## Abstract

**Background:**

Identifying preventable diets and environmental exposure is essential to ensuring the health of the aging population. This study evaluated the interaction effect between blood cadmium and ω-6 fatty acids intake on low cognitive performance in Americans.

**Method:**

The data of this cross-sectional study were obtained from the 2011–2012 and 2013–2014 National Health and Nutritional Examination Survey (NHANES). Cognitive performance was measured by the Consortium to Establish a Registry for Alzheimer’s Disease test, Animal Fluency Test, and Digit Symbol Substitution Test. Multivariate logistic regression models were used.

**Results:**

A total of 1,918 individuals were included, with 467 (24.35%) low cognitive performance. Compared with participants with normal-level blood cadmium, those with high-level blood cadmium had a higher risk of low cognitive performance [odds ratio (OR) was 1.558 with 95% confidence interval (CI): 1.144–2.123]. Low-level ω-6 fatty acids intake was positively associated with low cognitive performance [OR = 1.633 (95%CI: 1.094–2.436)] compared with normal-level intake. Moreover, there was a significant interaction between low-level ω-6 fatty acids intake and high-level blood cadmium on the risk of low cognitive performance (relative excess risk due to interaction: 0.570, 95%CI: 0.208-0.932; the attributable proportion of interaction: 0.219, 95%CI: 0.102‐0.336; synergy index: 1.552, 95%CI: 1.189‐2.027).

**Conclusions:**

There was a synergistic interaction between low-level ω-6 fatty acids intake and high-level blood cadmium on low cognitive performance. Low-level ω-6 fatty acids intake may amplify the adverse effects of long-term exposure to cadmium on cognitive performance. This may have a certain significance for the prevention of cognitive decline in the elderly.

**Supplementary information:**

The online version contains supplementary material available at 10.1186/s12877-022-02988-7.

## Background

The global population is rapidly aging due to the low birth rate and the extended lifespan [1–3]. In 2019, the proportion of elderly people over 65 years old accounted for about 9% in the world, and it will increase to 16% by 2050 [2]. The situation is more serious in the United States, where about 18% of Americans were 65 years of age or older in 2019 [3]. The elderly face many health threats. Among them, cognitive decline is a major killer that threatens the elderly following chronic diseases such as cardiovascular disease [1, 4]. Cognitive impairments associated with old age are expected to impose heavy social and economic burdens [5, 6]. The total cost of individuals with low cognitive performance in 2020 was estimated to be 305 billion dollars in the United States [5]. Therefore, detecting the preclinical manifestations of low cognitive performance as early as possible is important.

In addition to good living habits and proper physical exercise to prevent cognitive decline, the most important is to have a reasonable diet and supplement nutrition. Studies have reported that individuals with a Mediterranean diet could effectively reduce the risk of cognitive decline, which may be related to their high intake of unsaturated fatty acids [7]. Evidence showed that a high-level (ω-3): (ω-6) dietary intake ratio had a wide range of positive effects on health, especially the improvement of cognitive function [8]. Studies have pointed out that the intake of ω-6 fatty acids (important unsaturated fatty acids in the human brain) may be related to cognitive decline [9, 10]. In addition, the severe environmental situation has led to increased exposure of people to heavy metals [11]. Cadmium, heavy metal from the Earth’s crust, could cause cognitive dysfunction because it has long-term effects on the brain [12, 13]. Cadmium ion poisoning can cause hippocampal damage and cognitive impairment [14]. However, the interaction between ω-6 fatty acids intake and blood cadmium on low cognitive performance has not been widely reported.

Therefore, this cross-sectional study intended to identify the determinants of low cognitive performance and to explore the interaction between ω-6 fatty acids intake and blood cadmium on the risk of low cognitive performance based on the National Health and Nutrition Examination Survey (NHANES) database in the United States.

## Methods

### Study population

We analyzed the data from the 2011–2012 and 2013–2014 NHANES, a representative cross-sectional survey of all non-institutionalized civilian populations in the United States. The NHANES is a major project of the National Center for Health Statistics (NCHS), a part of the Centers for Disease Control and Prevention (CDC), and is responsible for compiling life and health statistics. The NHANES includes interviews, physical examinations, and laboratory assessments. The NCHS Ethics Review Committee granted ethical approval. All individuals provided written informed consent before participating in the study.

Cognitive data on 2,934 adults aged 60 years or older were extracted from the NHANES database. We excluded those with missing blood cadmium (*n* = 866), and ω-6 fatty acids intake (*n* = 150) information. Finally, a total of 1918 participants were included in this study.

### Outcome variable

The word learning and recall modules from the Consortium to Establish a Registry for Alzheimer’s Disease (CERAD) test, Animal Fluency Test, and Digit Symbol Substitution Test (DSST) were applied to assess cognitive performance [15, 16]. The CERAD Word Learning Subtest (CERAD W-L) was used to evaluate the immediate and delayed learning ability of new language information (memory subdomain) [17]. The CERAD test consists of three consecutive learning trials and one delayed recall. After learning the test, participants were asked to recall as many words from the learning experiment as possible. The score for each test ranged from 0 to 10 points, with 1 point for each correct answer. The total score of the four tests was the CERAD score. The Animal Fluency test was used to measure absolute language fluency. Participants were asked to answer as many animals as possible within a minute, and each answer was scored one point. The DSST tested sustained attention and working memory [18]. Participants were asked to match the numbers in the 133 boxes to the corresponding symbols within 120 s according to the example given. The score was the sum of the number of correct matches, and the maximum score was 133 points.

Actually, there was no gold standard for judging low cognitive performance by the CERAD, Animal Fluency, and DSST test, and we utilized the lowest quartile (the 25th percentile) of the combined scores of the three tests in the study group as the cut-off point, which was consistent with the methods used in the published literature [19].

### Explanatory variables

Diet recall interviews were conducted at the Mobile Examination Center (MEC) by trained interviewers using an automated data collection system to obtain ω-6 fatty acids [linoleic (18:2) and arachidonic (20:4)] intake through two 24-hour dietary recall interviews. At the end of the MEC diet interviews, the interviewers arranged for the subjects to have a telephone follow-up interview 3–10 days later. Average ω-6 fatty acids intake was calculated based on the U.S. Department of Agriculture’s Dietary Study Food and Nutrition Database [20]. In the NHANES 2011–2014, ω-6 fatty acids intake was calculated only from dietary intake, and supplement usage was not collected.

Blood samples were transported to laboratories across the United States and the blood cadmium was detected by these laboratories. The blood samples were collected by a phlebotomist at the MEC and processed into vials, which were then refrigerated or frozen for storage and transported to laboratories across the United States. The concentration of blood cadmium was determined by quadrupole inductively coupled plasma mass spectrometry (ICP-MS) technology. Please refer to the NHANES laboratory manual for the specific method of blood cadmium content detection [21].

ω-6 fatty acids intake and blood cadmium were categorical variables, ω-6 fatty acids below the 25th quantile was low-level ω-6 fatty acids intake, and blood cadmium above the 75th quantile was high-level blood cadmium. The cut points of ω-6 fatty acids and blood cadmium were consistent with methods used in the published literature [22, 23].

### Covariates

Sociodemographic information, lifestyle factors, medical history, and laboratory parameters were collected. Sociodemographic information included age, gender, race, marital status (married/widowed or divorced or separated/never married/living with a partner), educational level [below high school/high school graduate or General Educational Development (GED)/above high school], and annual household income (< 20,000 dollars/ ≥20,000 dollars).

Lifestyle factors included trouble sleeping, sleeping time, smoking, drinking, work activity, and recreational activity. Trouble sleeping was assessed by a question that has a doctor or other health professionals ever told him/her had trouble sleeping. Smoking was assessed by smoking at least 100 cigarettes in one’s entire life, and drinking was assessed by drinking at least 12 drinks of any type of alcoholic beverage in any one year (a drink means a 12 oz. beer, a 5 oz. glass of wine, or one and a half ounces of liquor). Work activity was divided into three categories: vigorous work activity, moderate work activity, and others [15, 16]. The recreational activity was divided into three categories: vigorous recreational activity; moderate recreational activity and others [15, 16].

Depression, hypertension, diabetes, stroke, congestive heart failure (CHF), coronary heart disease (CHD), and heart attack were assessed by asking participants, “Have you ever been told by a doctor or health professional that you have __?” Total cholesterol (TC), high-density lipoprotein (HDL), glycated hemoglobin (GHb), and 25-hydroxyvitamin D [25(OH)D] were all obtained by remote laboratory testing of participants’ blood. In addition, body mass index (BMI) was calculated by dividing the weight of the participant by the square of the height (kg/m^2^).

### Statistical analysis

WTMEC2YR, SDMVPSU, and SDMVSTRA from the NHANES database were used as weighted variables to perform weighted analysis on all data. WTMEC2YR was the two-year sample weighed. SDMVPSU was masked variance unit pseudo-PSU variable for variance estimation. SDMVSTRA was masked variance unit pseudo-stratum variable for variance estimation. Measurement data were all normally distributed after weighting, and normality was assessed by the Kolmogorov-Smirnov test [24]. Normally distributed data were described by Mean [standard error (S.E)], and the t-test was used for the comparison between groups. Counting data were shown as the number of cases and the composition ratio [n (%)], and the comparison between groups was performed by the χ2 test or Fisher’s exact test. First, we conducted univariate analysis, and then multivariate logistic regression analysis was performed including the statistically different variables to explore whether low-level ω-6 fatty acids intake and high-level blood cadmium were associated with low cognitive performance. Model 1 was not adjusted for any confounders. Age, gender, and BMI were adjusted in Model 2, and in addition to variables adjusted by Model 2, variables that were statistically significant in the univariate analysis [race, marital status, educational level, annual household income, drinking, work activity, recreational activity, depression, hypertension, diabetes, stroke, CHF, heart attack, TC, GHb, and 25(OH)D] were adjusted in Model 3. Last, the interaction model was constructed to study whether the interaction existed. The synergistic interaction between low-level ω-6 fatty acids intake and high-level blood cadmium in association with low cognitive performance was measured by whether the estimated joint effect of two factors was greater than the sum of the independent effect of low-level ω-6 fatty acids intake and high-level blood cadmium. Relative excess risk due to interaction (RERI), the attributable proportion of interaction (AP), and synergy index (S) were utilized to assess synergistic interaction. When the confidence interval of RERI and AP contained 0 and the confidence interval of S contained 1, there was no synergistic interaction.

All statistical tests were two-sided and completed using SAS v. 9.4 (SAS Institute, Cary, North Carolina) statistical analysis software. *P* < 0.05 was considered statistically significant.

## Results

### Characteristics of the study population

A total of 1,918 samples were finally included in the study. The gender distribution was relatively equal, with 954 (46.45%) males and 964 (53.55%) females. Of these participants, 946 (81.49%) were non-Hispanic white, followed by other races (*n* = 365, 8.12%), non-Hispanic black (*n* = 455, 7.46%), and Mexican Americans (*n* = 152, 2.92%). Most participants was married [*n* = 1067 (63.92%)], and the highest degree of education was above high school [*n* = 976 (61.56%)]. What’s more, 495 (22.23%) of the participants had high-level blood cadmium, and the mean (S.E.) of blood cadmium was 0.49 (0.02) µg/L. Participants with low-level ω-6 fatty acids intake were 480 (22.22%). The descriptive characteristics of the included individuals are shown in Table 1. We compared the characteristics of the population included in this analysis with those of the population excluded due to missing data. We observed that the characteristics of the samples included in this analysis were similar to those of individuals with missing data (*P* > 0.05), indicating that our subsequent conclusions were reliable and stable (Supplement Table 1).

**Table 1:** Characteristics of the study population

Characteristics	Total (*n* = 1,918)	Cognitive performance		Statistics	*P*
		Low cognitive performance (*n* = 467)	Normal cognitive performance (*n* = 1,451)		
Age, years, n (%)				χ^2^ = 9.584	0.008
60–70	1,046 (56.41)	785 (57.69)	261 (47.93)		
70–80	574 (29.94)	435 (29.58)	139 (32.33)		
≥ 80	298 (13.65)	231 (12.74)	67 (19.73)		
Gender, n (%)				χ^2^ = 0.350	0.554
Male	954 (46.45)	687 (46.15)	267 (48.48)		
Female	964 (53.55)	764 (53.85)	200 (51.52)		
BMI, kg/m^2^, n (%)				χ^2^ = 7.156	0.067
<18.5	27 (1.19)	15 (0.88)	12 (3.27)		
18.5–25	494 (25.49)	369 (24.85)	125 (29.75)		
25–30	653 (35.83)	499 (36.60)	154 (30.75)		
≥30	744 (37.48)	568 (37.67)	176 (36.24)		
Race, n (%)				χ^2^ = 195.589	< 0.001
Mexican American	152 (2.92)	91 (1.99)	61 (9.08)		
Non-Hispanic White	946 (81.49)	831 (85.77)	115 (53.05)		
Non-Hispanic Black	455 (7.46)	283 (5.45)	172 (20.88)		
Other race	365 (8.12)	246 (6.79)	119 (16.99)		
Marital status, n (%)				χ^2^ = 25.228	< 0.001
Married	1,067 (63.92)	842 (66.45)	225 (47.11)		
Widowed/Divorced/Separated	680 (28.74)	486 (26.76)	194 (41.92)		
Never married	117 (4.34)	84 (4.01)	33 (6.54)		
Living with partner	54 (3.00)	39 (2.79)	15 (4.44)		
Educational level, n (%)				χ^2^ = 212.195	< 0.001
Below high school	485 (15.59)	226 (11.05)	259 (45.81)		
High school/GED	457 (22.85)	347 (21.92)	110 (29.02)		
Above high school	976 (61.56)	878 (67.03)	98 (25.17)		
Annual household income, dollars, n (%)				χ^2^ = 92.482	< 0.001
<20,000	512 (16.49)	299 (12.73)	213 (41.47)		
≥20,000	1,406 (83.51)	1,152 (87.27)	254 (58.53)		
Trouble sleeping, n (%)	221 (11.53)	172 (11.59)	49 (11.14)	χ^2^ = 0.044	0.834
Sleeping time, hours, Mean (S.E)	7.12 (0.03)	7.14 (0.04)	7.02 (0.08)	t = 1.210	0.237
Smoking, n (%)	975 (50.99)	715 (50.18)	260 (56.40)	χ^2^ = 3.709	0.054
Drinking, n (%)	590 (26.09)	429 (24.30)	161 (38.01)	χ^2^ = 22.038	< 0.001
Work activity, n (%)				χ^2^ = 15.971	< 0.001
Vigorous	216 (13.43)	179 (14.27)	37 (7.80)		
Moderate	399 (23.02)	334 (24.30)	65 (14.48)		
Other	1,303 (63.56)	938 (61.43)	365 (77.72)		
Recreational activity, n (%)				χ^2^ = 61.881	< 0.001
Vigorous	165 (11.45)	153 (12.90)	12 (1.78)		
Moderate	625 (33.63)	502 (35.02)	123 (24.40)		
Other	1,128 (54.92)	796 (52.08)	332 (73.82)		
Depression, n (%)	148 (5.64)	82 (4.38)	66 (14.03)	χ^2^ = 27.250	< 0.001
Hypertension, n (%)	1,177 (56.69)	872 (55.15)	305 (66.94)	χ^2^ = 10.486	0.001
Diabetes, n (%)	458 (19.61)	300 (17.36)	158 (34.62)	χ^2^ = 22.682	< 0.001
Stroke, n (%)	129 (6.01)	79 (5.25)	50 (11.01)	χ^2^ = 9.044	0.003
CHF, n (%)	133 (6.82)	82 (5.80)	51 (13.61)	χ^2^ = 21.130	< 0.001
CHD, n (%)	167 (8.25)	125 (8.18)	42 (8.74)	χ^2^ = 0.092	0.762
Heart attack, n (%)	166 (8.52)	117 (7.87)	49 (12.84)	χ^2^ = 6.794	0.009
TC, mg/dL Mean (S.E)	195.04 (1.49)	196.32 (1.58)	186.52 (2.43)	*t* = 3.880	< 0.001
HDL, mg/dL, Mean (S.E)	55.99 (0.88)	56.28 (0.97)	54.04 (1.18)	*t* = 1.600	0.119
GHb, %, Mean (S.E)	5.94 (0.03)	5.88 (0.04)	6.35 (0.12)	*t*=-3.760	< 0.001
25(OH)D, nmol/L, Mean (S.E)	82.16 (1.30)	83.52 (1.29)	73.09 (2.65)	*t* = 4.310	< 0.001
Blood cadmium, µg/L, Mean (S.E)	0.49 (0.02)	0.47 (0.02)	0.63 (0.05)	*t*=-2.890	0.007
Blood cadmium, µg/L, n (%)				χ^2^ = 26.958	< 0.001
Normal-level (< 0.60)	1,423 (77.77)	1,112 (79.68)	311 (65.07)		
High-level (≥ 0.60)	495 (22.23)	339 (20.32)	156 (34.93)		
Total ω-6 fatty acids intake, mg/kcal/day, Mean (S.E)	8.18 (0.08)	8.30 (0.09)	7.34 (0.17)	*t* = 4.470	< 0.001
Total ω-6 fatty acids intake, mg/kcal/day, n (%)				χ^2^ = 9.222	0.002
Normal-level (≥ 5.92)	1438 (77.78)	1,111 (79.11)	327 (68.89)		
Low-level (< 5.92)	480 (22.22)	340 (20.89)	140 (31.11)		

*S.E* standard error, *BMI* body mass index, *GED* general educational development, *CHF* congestive heart failure, *CHD* coronary heart disease, *TC* total cholesterol, *HDL* high-density lipoprotein, *GHb* glycated hemoglobin, *25(OH)D* 25-hydroxyvitamin D

## Comparison of the normal and low cognitive performance groups

In Table 1, the distribution of age, race, marital status, educational level, annual household income, drinking, work activity, recreational activity, depression, hypertension, diabetes, stroke, CHF and heart attack, the level of GHb, 25(OH)D, blood cadmium, and ω-6 fatty acids intake in the low cognitive performance group were different from those in the normal cognitive performance group, and the differences were statistically significant (*P* < 0.05).

### Independent association of ω-6 fatty acids intake and blood cadmium with low cognitive performance

Compared with participants who had normal-level blood cadmium, those who had high-level blood cadmium were associated with the greater risk of low cognitive performance [odds ratio (OR) with 95% confidence interval (CI) of 1.558 (1.144–2.123)], after adjusting for age, gender, BMI, race, marital status, educational level, annual household income, drinking, work activity, recreational activity, depression, hypertension, diabetes, stroke, CHF, heart attack, TC, GHb, and 25(OH)D. The risk of low cognitive performance in participants with low-level ω-6 fatty acids intake was 1.633 times that in those with normal-level ω-6 fatty acids intake (OR = 1.633, 95%CI: 1.094–2.436) in Model 3. The detailed results of the multivariable logistic regression models of an independent association of ω-6 fatty acids intake and blood cadmium with low cognitive performance are shown in Table 2.

**Table 2:** Independent association of ω-6 fatty acids intake and blood cadmium on low cognitive performance

Variables	Model 1			Model 2			Model 3	
	OR (95%CI)	*P*		OR (95%CI)	*P*		OR (95%CI)	*P*
Blood cadmium								
Normal-level	Ref			Ref			Ref	
High-level	2.104 (1.547–2.863)	< 0.001		2.006 (1.516–2.653)	< 0.001		1.558 (1.144–2.123)	0.006
Total ω-6 fatty acids intake								
Normal-level	Ref			Ref			Ref	
Low-level	1.711(1.195–2.449)	0.005		1.694 (1.177–2.439)	0.006		1.633 (1.094–2.436)	0.018

*OR* odds ratio, *CI* confidence interval, *Ref* reference

Model 1: Univariable logistic regression analysis

Model 2: Multivariate logistic regression analysis with adjustment for age, gender, and BMI;

Model 3: Multivariate logistic regression analysis with adjustment for age, gender, BMI, race, marital status, educational level, annual household income, drinking, work activity, recreational activities, depression, hypertension, diabetes, stroke, CHF, heart attack, TC, GHb, 25(OH)D

### Interaction between ω-6 fatty acids intake and blood cadmium on low cognitive performance

**Fig. 1:**
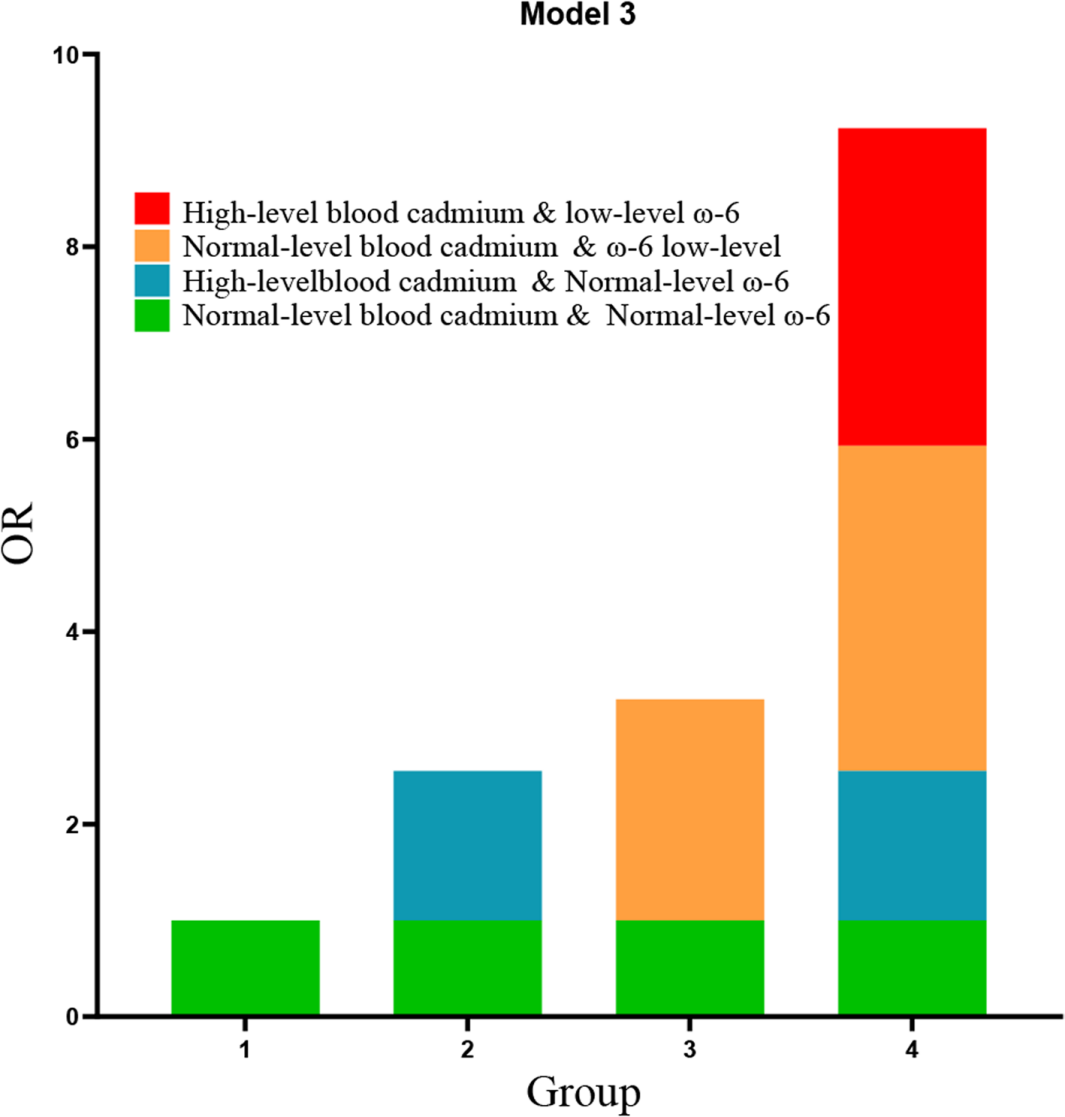
Interaction between ω-6 fatty acids intake and blood cadmium in Model 3

Results in Table 3 showed that the interaction indicators RERI was 0.570 (95%CI: 0.208–0.932), AP was 0.219 (95%CI: 0.102–0.336) and S was 1.552 (95%CI: 1.189–2.027), indicating that the interaction of low-level ω-6 fatty acids intake and high-level blood cadmium on low cognition was statistically significant, which was a synergistic effect. Among them, after adjusting for the variables with differences in univariate analysis, AP was 0.219, indicating that 21.9% of low cognitive performance were caused by the interaction between low-level ω-6 fatty acids intake and high-level blood cadmium in the sample of this study. Figure 1 provides a visual comparison of the interaction effect of low-level ω-6 fatty acids intake and high-level blood cadmium on low cognitive performance by OR value.

**Table 3:** Logistic regression analyses of the interaction between ω-6 fatty acids intake and blood cadmium on low cognitive performance

Total fatty acids intake	Blood cadmium	Model 1		Model 2		Model 3	
		OR (95%CI)	*P*	OR (95%CI)	*P*	OR (95%CI)	*P*
ω-6 fatty acids							
Normal-level	Normal-level	Ref		Ref		Ref	
Normal-level	High-level	2.069 (1.535–2.788)	< 0.001	1.955 (1.466–2.605)	< 0.001	1.484 (1.083–2.037)	0.011
Low-level	Normal-level	1.666 (1.149–2.414)	0.005	1.637 (1.124–2.383)	0.012	1.548 (1.005–2.386)	0.039
Low-level	High-level	3.403 (1.814–6.384)	< 0.001	3.237 (1.743–6.011)	< 0.001	2.602 (1.310–5.169)	0.005
RERI (95%CI)		0.668 (0.240–1.096)		0.654 (0.248–1.060)		0.570 (0.208–0.932)	
AP (95%CI)		0.196 (0.089–0.304)		0.202 (0.095–0.309)		0.219 (0.102–0.336)	
S (95%CI)		1.385 (1.137–1.687)		1.413 (1.154–1.730)		1.552 (1.189–2.027)	

*OR* odds ratio, *CI* confidence interval, *Ref* reference, *RERI* relative excess risk due to interaction, *AP* attributable proportion of interaction, *S* synergy index

Model 1: Univariable logistic regression analysis

Model 2: Multivariate logistic regression analysis with adjustment for age, gender and BMI

Model 3: Multivariate logistic regression analysis with adjustment for age, gender, BMI, race, marital status, educational level, annual household income, drinking, work activity, recreational activities, depression, hypertension, diabetes, stroke, CHF, heart attack, TC, GHb, 25(OH)D

## Discussion

In this large, nationally representative sample of adults aged 60 years or older among the U.S. population, we found that there was a synergistic interaction between low-level ω-6 fatty acids intake and high-level blood cadmium on low cognitive performance. The results suggested that we could improve diet to enhance the mental fitness of the elderly and pay attention to reducing cadmium exposure.

Cognitive decline is a complex and gradual process that goes through different stages of evolution: normal cognition, memory impairment, mild cognitive impairment, and dementia [25]. The onset usually takes a long time, so prevention is particularly important during treatment [25, 26]. It is an effective way to improve the physical condition of the elderly and improve cognitive decline by improving the diet [27]. In the present study, we found that low-level ω-6 fatty acids intake might be associated with low cognitive performance after controlling for confounders, including age, gender, BMI, race, marital status, educational level, income, drinking, work activity, recreational activity, depression, hypertension, diabetes, stroke, CHF, heart attack, TC, GHb, and 25(OH)D, which was consistent with a study conducted by Xue Dong et al. [8]. The authors also used the data from the NHANES 2011–2014, but the adjusted confounders were slightly different from ours, and their conclusions were drawn by adjusting for age, gender, race, educational level, marital status, income, BMI, recreational activity, work activity, drinking status, hypertension, diabetes, and stroke [8]. We also adjusted for some additional laboratory indicators. Moreover, in other study populations from the U.S., the results identified that ω-6 fatty acids as nutrient biomarkers were associated with more favorable functional efficiency in the aging brain [28]. Increasing evidence indicated that ω-6 fatty acids would be of benefit to the brain in aging adults [29]. Although the intake of ω-6 fatty acids in the diet has a positive impact on cognitive outcomes by enhancing the nervous system, for the intake of ω-6 fatty acids, the ratio of ω-6: ω-3 is important [8, 28, 30]. Further research is needed to study the ratio value to further promote brain and cognitive health.

The evidence for the relationship between cadmium and cognitive performance in the elderly population was limited. In this study, we found a significantly positive association between high-level blood cadmium and low cognitive performance of U.S. adults aged over 60 years from the NHANES 2011–2014, and the association did not change after controlling for potential confounders. Similarly, a study using the same database and time span as ours suggested that increased blood cadmium was significantly associated with worse cognitive performance in adults aged 60 years or older in the U.S. [13]. However, another study did not find the association between cadmium and cognitive functioning using the data from the NHANES 1999–2002 after adjustment for race, age, sex, poverty income ratio, education, and smoking status [30]. The main reason may be that the selected study population was different. The population of the study was the elderly in the NHANES 1999–2002, while that of our study was the elderly in the NHANES 2011–2014. The average level of blood cadmium has increased, leading to different results. The different results may be caused by the control of different confounders, the different study designs used, or the different effects of cadmium in the research design. In addition, the findings of a prospective cohort study in southwestern and eastern China suggested that higher cadmium exposure was associated with greater cognitive decline in Chinese adults aged 65 years or older [31]. The negative correlation between blood cadmium and cognitive performance was of great significance for proposing strategies to delay the decline of cognitive performance in the elderly. Healthy diet and behavior could change exposure to cadmium, as cadmium is a cumulative poison, mainly from food and tobacco smoke [32]. The changes may improve the cognitive performance of adults over the age of 60 years.

The mechanism underlying our finding that there was a synergistic interaction between lower ω-6 fatty acids and higher blood cadmium on greater cognitive decline may be supported by studies of acetylcholine release or inflammation. Arachidonic, a member of the ω-6 series, could enhance acetylcholine release in the brain, which may be beneficial to cognitive performance [29, 33]. Studies have illustrated that cadmium exposure could increase the activity of acetylcholinesterase, causing acetylcholine to be hydrolyzed and reducing its concentration [34]. Decreased acetylcholine release was related to low cognitive performance [35]. In addition, studies have shown that cadmium induced the formation of reactive oxygen species (ROS) [32]. Excessive ROS may cause inflammation and lead to neuronal damage and death ultimately [32, 36]. ω-6 and ω-3 fatty acids exerted anti-inflammatory properties through a competitive relationship [37]. Studies on healthy adults have found that increasing the intake of ω-6 fatty acids did not increase the concentration of inflammatory markers [38]. Also, studies indicated that arachidonic and linoleic may be related to inflammation reduction [38, 39]. The mechanism of ω-6 fatty acids intake or blood cadmium on low cognitive performance was still unclear. We only presented the possible mechanism of interaction between ω-6 fatty acids intake and blood cadmium. Therefore, further research was required to explore the relationship between blood cadmium and ω-6 fatty acids intake on cognitive performance. Nuts (sunflower, pumpkin seeds, walnuts) and vegetable (corn, sunflower, and soybean) oils are rich in ω-6 fatty acids [40]. Shellfish (oysters, bivalve mollusks, etc.) and offal products contain high concentrations of cadmium [32]. The cadmium content of plant foods depends on the degree of soil contamination, and is generally of a higher concentration than that of meat, eggs, milk, and dairy products [32]. Vegetarians and shellfish consumers may have higher cadmium intakes than omnivores. The elderly should pay attention to dietary diversity, which may have certain benefits on cognitive function.

The strengths of this study were as follows. Firstly, our research sample was representative, including a relatively large sample of senior citizens in the four main races. Secondly, there were no studies about the interaction between ω-6 fatty acids intake and blood cadmium on low cognitive performance, and our research showed that there may be a synergistic effect between low-level ω-6 fatty acids intake and high-level blood cadmium. Thirdly, the database we used evaluated the cognitive performance of the elderly through three objective cognitive assessment methods, the CERAD, Animal fluency, and DSST, which were carried out in a private and standardized environment. It was more similar to a clinical rather than a household setting. However, a few limitations should be noted in our study. First, due to the cross-sectional nature of the NHANES, the confounders of unmeasured data could not be determined although confounders were excluded as much as possible in this study. Besides, there was no way to determine whether the measured low cognitive performance represented a change in an individual’s cognitive performance. Second, the cognitive performance tests did not cover all domains of cognition. Adults who perform well in one cognitive test may not perform well in another. But these three cognitive tests existed for ease of management and use in other surveys. Third, the dietary data in the NHANES were obtained from two 24-hour recall interviews, which may be biased in information and could not accurately reflect daily intake of an individual.

## Conclusions

Our results indicated that low-level ω-6 fatty acids intake and high-level blood cadmium had a synergistic interaction on low cognitive performance, suggesting that the elderly could appropriately increase ω-6 fatty acids intake and reduce their exposure to cadmium. These findings may provide epidemiological evidence for the independent association of ω-6 fatty acids intake and blood cadmium with low cognitive performance, and the interaction association between ω-6 fatty acids intake and blood cadmium on low cognitive performance.

## Supplementary Information

**Additional file 1.**

## Data Availability

The datasets used and/or analyzed during the current study are available from the corresponding author on reasonable request.

## Abbreviations

NHANES: National Health and Nutrition Examination Survey
NCHS: National Center for Health Statistics
CDC: Centers for Disease Control and Prevention
CERAD: Consortium to Establish a Registry for Alzheimer’s Disease
DSST: Digit Symbol Substitution Test
CERAD W-L: CERAD Word Learning Subtest
MEC: mobile examination center
ICP-MS: inductively coupled plasma mass spectrometry
GED: General Educational Development
CHF: congestive heart failure
CHD: coronary heart disease
TC: Total cholesterol
HDL: high-density lipoprotein
25(OH)D: 25-hydroxyvitamin D
BMI: body mass index
S.E: standard error
n (%): composition ratio
RERI: Relative excess risk due to interaction
AP: attributable proportion of interaction
S: synergy index
