# Supplementary material for: Interaction between ω-6 fatty acids intake and blood cadmium on the risk of low cognitive performance in older adults from National Health and Nutrition Examination Survey (NHANES) 2011–2014
Source: BMC Geriatr. 2022 Apr 7;22:292. doi: 10.1186/s12877-022-02988-7 (PMC8988388; doi:10.1186/s12877-022-02988-7)
Supplement: Supplementary file 1 — Additional file 1. [file 12877_2022_2988_MOESM1_ESM.docx]

Supplement table 1 Sensitivity analysis of interpolation data

| Characteristics | Missing data (%) | After interpolation | Before interpolation | Statistics | *P* |
| --- | --- | --- | --- | --- | --- |
| Age, years | 0.0 |  |  |  |  |
| Gender | 0.0 |  |  |  |  |
| Race | 0.0 |  |  |  |  |
| Depression | 0.0 |  |  |  |  |
| Hypertension | 0.0 |  |  |  |  |
| Stroke | 0.0 |  |  |  |  |
| TC, mg/dL | 0.0 |  |  |  |  |
| GHb, % | 0.0 |  |  |  |  |
| Cognitive | 0.0 |  |  |  |  |
| Total ω-6 fatty acids intake, mg/kcal/day | 0.0 |  |  |  |  |
| Blood cadmium, μg/L | 0.0 |  |  |  |  |
| BMI, kg/m^2^, Mean (S.E) | 5.3 | 29.08 (0.24) | 29.09 (0.25) | t=-0.20 | 0.842 |
| Diabetes, n (%) | 4.4 |  |  | χ^2^=0.992 | 0.319 |
| No |  | 458 (19.61) | 430 (19.36) |  |  |
| Yes |  | 1,460 (80.39) | 1,404 (80.64) |  |  |
| Annual household income, dollars, n (%) | 4.2 |  |  | χ^2^=0.715 | 0.398 |
| <20,000 |  | 512 (26.69) | 489 (25.50) |  |  |
| ≥20,000 |  | 1,406 (73.31) | 1,429 (74.50) |  |  |
| TC, mg/dL, Mean (S.E) | 2.6 | 195.04 (1.49) | 195.12 (1.55) | t=-0.47 | 0.639 |
| HDL, mg/dL Mean (S.E) | 2.6 | 55.99 (0.88) | 55.91 (0.90) | t=0.92 | 0.362 |
| 25(OH)D, nmol/L, Mean (S.E) | 1.6 | 82.16 (1.30) | 81.99 (1.35) | t=1.62 | 0.115 |
| Drinking, n (%) | 0.7 |  |  | χ^2^=0.028 | 0.868 |
| No |  | 1,328 (73.91) | 1,319 (73.90) |  |  |
| Yes |  | 590 (26.09) | 585 (26.10) |  |  |
| CHF, n (%) | 0.5 |  |  | χ^2^=0.585 | 0.444 |
| No |  | 1,785 (93.18) | 1,777 (93.20) |  |  |
| Yes |  | 133 (6.82) | 131 (6.80) |  |  |
| CHD, n (%) | 0.5 |  |  | χ^2^=0.962 | 0.327 |
| No |  | 1,751 (91.75) | 1,744 (91.78) |  |  |
| Yes |  | 167 (8.25) | 164 (8.22) |  |  |
| GHb, Mean (S.E) | 0.3 | 5.94 (0.03) | 5.94 (0.03) | t=0.48 | 0.636 |
| Stoke, n (%) | 0.2 |  |  | χ^2^=2.407 | 0.121 |
| No |  | 1,789 (93.99) | 1,785 (93.98) |  |  |
| Yes |  | 129 (6.01) | 129 (6.02) |  |  |
| Trouble sleeping, n (%) | 0.2 |  |  | χ^2^=1.703 | 0.192 |
| No |  | 1,697 (88.47) | 1,694 (88.46) |  |  |
| Yes |  | 221 (11.53) | 221 (11.54) |  |  |
| Sleeping time, hours, Mean (S.E) | 0.2 | 7.12 (0.03) | 7.12 (0.03) | t=1.72 | 0.096 |
| Hypertension, n (%) | 0.1 |  |  | χ2=0.256 | 0.613 |
| No |  | 741 (43.31) | 740 (43.34) |  |  |
| Yes |  | 1,177 (56.69) | 1,176 (56.66) |  |  |
| Heart attack, n (%) | 0.1 |  |  | χ^2^=1.890 | 0.169 |
| No |  | 1,752 (91.48) | 1,750 (91.48) |  |  |
| Yes |  | 166 (8.52) | 166 (8.52) |  |  |
| Marital status, n (%) | 0.1 |  |  | χ^2^=3.363 | 0.339 |
| Married |  | 1,067 (63.92) | 1,067 (63.93) |  |  |
| Widowed/Divorced/Separated |  | 680 (28.74) | 680 (28.74) |  |  |
| Never married |  | 117 (4.34) | 116 (4.32) |  |  |
| Living with partner |  | 54 (3.00) | 54 (3.00) |  |  |
| Educational level, n (%) | 0.1 |  |  | χ^2^=1.943 | 0.378 |
| Below high school |  | 485 (15.59) | 485 (15.59) |  |  |
| High school/GED |  | 457 (22.85) | 457 (22.85) |  |  |
| Above high school |  | 976 (61.56) | 975 (61.56) |  |  |
| Smoking, n (%) | 0.1 |  |  | χ^2^=1.051 | 0.305 |
| No |  | 943 (49.01) | 942 (49.00) |  |  |
| Yes |  | 975 (50.99) | 975 (51.00) |  |  |

S.E: standard error; BMI: body mass index; GED: general educational development; CHF: congestive heart failure; CHD: coronary heart disease; TC: total cholesterol; HDL: high-density lipoprotein; GHb: glycated hemoglobin; 25(OH)D: 25-hydroxyvitamin D
